# Factors associated with genital prolapse to Saint Joseph Hospital of Kinshasa

**DOI:** 10.11604/pamj.2021.40.234.30529

**Published:** 2021-12-16

**Authors:** Antoine Tshimbundu Kayembe, Charles Didier Kitenge Kia Kayembe, Jean-Patrick Kamba Bebele, Rahma Rachid Tozin

**Affiliations:** 1 Department of Gynaecology and Obstetrics, Faculty of Medicine, University Notre-Dame of Kasayi, Central Kasaï, Democratic Republic of the Congo,; 2 Department of Gynaecology and Obstetrics, Faculty of Medicine, University of Kinshasa, Kinshasa, Democratic Republic of the Congo

**Keywords:** Genital prolapse, factors associated, Saint Joseph hospital, Kinshasa

## Abstract

**Introduction:**

the aim of this study was to identify factors associated with genital prolapse in the gynecology and obstetrics service of Saint Joseph hospital of Kinshasa

**Methods:**

this was a retrospective case-control study conducted from 148 medical files of patients admitted in the gynecology and obstetrics service of Saint Joseph hospital from January 1, 2008 to December 31, 2017. It was based on the non-probabilistic sampling of suitability for cases selection. The T-student test, Chi-test and logistic regression were used in statistical analyses

**Results:**

five factors independently associated with genital prolapse were identifying: obesity with BMI≥30Kg/m^2^(OR: 3.770, 95% CI: 1.040-9.250; p=0.001), menopause (OR: 1.910, 95% CI: 1.090-10.930; p=0.001), fœtal macrosomia (OR: 4.290, 95% CI: 3.320-5.550; p=0.000), vaginal delivery (OR: 2.070, 95% CI: 1.010-5.210; p=0.006) and perineal tears (OR: 1.510, 95% CI: 1.250-1.910; p=0.000).

**Conclusion:**

these factors independently associated with genital prolapse can be used for screening of high-risk women in gynecological and obstetrical consultations in order to improve the treatment of genital prolapse in our milieu.

## Introduction

The genital prolapse constitutes a frequent reason for medical consultations and a growing indication of surgery in gynecology and urology units [1,2]. Considering various factors notably the increased life expectancy, the number of patients who experience genital prolapse will double in the next decade [3]. The worldwide prevalence of genital prolapse varies from 2.9% to 97.7% depending on the method used for the study. It is estimated from 2.9% to 11.4% when the method used is a survey [4-13] and from 31.8% to 97.7% when clinical examination with the pelvic organ prolapses quantification (POPQ) is performed [14-21]. In Asia and Africa, this prevalence is not known due to the lack of survey and studies in the general population [21]. In the Democratic Republic of Congo, this prevalence is not known and data to estimate its incidence are nonexistent [21]. The genital prolapse is a dynamic disease which can worsen or recede especially among pregnant women in the postpartum period [14,22]. The recurrence risk is high after surgical treatment [22,23]. It causes several urinary, digestive and genital troubles which hamper the quality of life of patients [4,9,22]. Risk factors involved in its occurrence are of two orders: modifiable risk factors (obesity: body weight ≥90 Kg or BMI ≥30 Kg/m^2^, vaginal delivery, parity, smoking, foetal macrosomia, perineal tear, profession and low social and economic level) [22,24-28]; and no modifiable risk factors (age, white race, menopause, chronic obstructive pulmonary diseases, rachidian anomaly, personal and familial history of genital prolapse, pelvic surgery and chronic constipation) [22,24-27,29].

The lack of data about factors associated with genital prolapse in our milieu highlighted the need to conduct the present study with aims at identifying factors associated with genital prolapse in gynecology and obstetrics service (GOS) of Saint Joseph Hospital (SJH) of Kinshasa.

## Methods

**Study design and setting:** this was a case-control study in which cases were patients who suffered from genital prolapse and controls were patients who suffered from other disease in GOS of SJH of Kinshasa from January 1, 2008 to December 31, 2017. The GOS of SJH of Kinshasa was chosen because of the presence of formed medical staff, the frequent contact with patients who suffered from genital prolapse, and a free management through the fistula care program.

**Study population:** we used medical files of patients who suffered from genital prolapse (cases) and from other disease (controls), treated in GOS of SJH of Kinshasa from January 1, 2008 to December 31, 2017 and paired according to the parity and the age. All medical files not found and those that contained less than 50% of studied variables were excluded. One hundred and forty-eight available cases and two hundred and ninety-six control have been included and thirteen cases were excluded because their medical files have not been found (either in total one hundred sixty-one cases of genital prolapse registered).

**Sampling:** our sampling was non-probabilistic of suitability for cases selection. The sample size was calculated using the following formula:


$$n\geq \frac{\left(1+\frac{1}{c}\right){\left(Z_{\alpha }+Z_{1-\beta }\right)}^{2}P(1-P)}{{\left(P_{0}-P_{1}\right)}^{2}}$$

and T=nxc [23-26] where: c: number of controls by cases (c=2), n: sample size, P: proportion in two groups (P=0.65).


$$P=\frac{P_{1}+cP_{0}}{1+c}$$

P_0_: controls proportion; (P_0_=0.60), P_1_: cases proportion (P_1_=0,75).


$$P_{1}=\frac{P_{0}xOR}{1+P_{0}(OR-1)}$$

T: total number of controls, Z_α_: Z-value for the risk of first species (1.645), α: risk of first species (0.05), Z_1-β_: Z-value for a power 1-β (1.282), 1-β: power wished (0.9) [23-26]. The calculated sample size was superior to 128 cases. One case was paired with two controls. Pairing criteria was the age and the parity because they were confounding factors and responded to pairing criteria.

**Data collection:** data were collected from registries of GOS and operating rooms, medical files of patients who suffered from genital prolapse (cases) and from other disease (control) at SJH and the data collection record. Variables for study were age of patients, weight, height, BMI, parity, menopausal state, vaginal delivery and their number, smoking, antecedent of foetal macrosomia, chronic pulmonary disease, perineal tears, personal and familial history for genital prolapse, pelvic surgery and spinal anomaly.

**Statistical analysis:** all statistical analyses were performed using SPSS (Statistical Package for Social Sciences) software version 20. The T-student test and the Chi-square test were used to compare averages and proportions between groups respectively. The univariable logistic regression analysis was used to evaluate the strength of association between observed factors and genital prolapse´s appearance. The multivariable logistic regression analysis was used to identify factors associated with genital prolapse among variable with p-value of less than 0.2 in the univariable analysis. A p-value <0.05 was considered statistically significant.

**Ethical considerations:** principles of medical ethics and documentary studies rules were respected: data were collected confidentially and treated anonymously.

## Results

**Frequency of genital prolapse:** we registered 161 cases of genital prolapse out of 13957 patients in GOS of SJH, resulting in an overall frequency of 1.2%. We remind that 148 available cases have been included and 13 excluded because their medical files have not been found or were not available (in total 161 cases for genital prolapse).

**Factors associated with genital prolapse:** risk factors of which the proportion was significantly superior in the group of cases compared to this one of control are multiparity (parity≥4), obesity with body weight ≥90kg, obesity with BMI≥30kg/m^2^, menopause, foetal macrosomia, pelvic tear, vaginal delivery, vaginal delivery number≥4, genital prolapses surgery (Table 1). Univariable analyses allowed us to note a significant association between genital prolapse´s occurrence and following factors: multiparity, obesity with body weight ≥90kg (obesity 1), obesity with BMI ≥30kg/m^2^ (obesity 2), menopause, fœtal macrosomia, pelvic or perineal tears, vaginal delivery and vaginal delivery number ≥4 (Table 2). Multivariable analyses identified the obesity with BMI≥30Kg/m^2^ (OR: 3.770, 95% CI: 1.040-9.250; p=0.001), the menopause (OR: 1.910, 95% CI: 1.090-10.930; p=0.001), the fœtal macrosomia (OR: 4.290, 95% CI: 3.320-5.550; p=0.000), the vaginal delivery (OR: 2.070, 95% CI: 1.010-5.210; p=0.006) and perineal tears (OR: 1.510, 95% CI: 1.250-1.910; p=0.000) as factors independently associated with genital prolapse (Table 2).

**Table 1:** factors associated with genital prolapse

Risk factors	Cases		Control		Total	
Age≥40 ans	78	52.70%	175	59.50%	254	57.30%
Multiparity	125	84.50%	138	46.70%	263	59.30%
Body weight	30	20.30%	2	0.70%	32	7.20%
Obesity (BMI ≥ 30Kg/m^2^)	100	67.60%	4	1.40%	104	23.40%
Menopause	57	38.50%	76	25.70%	133	30.00%
Fœtal macrosomia	120	82.20%	5	2.30%	125	34.00%
Vaginal delivery	147	99.30%	210	70.90%	357	80.40%
Vaginal delivery number ≥4	121	81.80%	125	42.20%	246	55.40%
pelvic ou perineal tear	136	92.50%	9	4.10%	145	39.20%
familial history for genital prolapse	0	0.00%	0	0.00%	0	0.00%
Personal history for genital prolapse	45	30.40%	0	0.00%	45	10,20%
Surgical history for genital prolapse	26	66.70%	0	0.00%	26	41.93%
Smoking	0	0.00%	0	0.00%	0	0.00%
chronic pulmonary diseases	33	22.30%	3	1.00%	36	8.11%
Spinal anomaly	0	0.00%	0	0.00%	0	0.00%

This table compares the difference between two groups (cases and control). It allows us to identify risk factors of which the proportion was superior, statistically significant, in cases group compared to this one of controls.

**Table 2:** factors independently associated with genital prolapse

	Univariable analysis		Multivariable analysis	
	OR (95% CI)	p-value	OR (95% CI)	p-value
Multiparity (reference: no)	6.220 (3.770-10.260)	<0.001	1.260 (0.040 - 34.980)	0.893
Body weight (reference: no)	3.740 (1.870-15.880)	<0.001	3.020 (0.0630 - 14.470)	0.576
Obesity (reference: no)	1.520 (1.234-4.320)	<0.001	3.110 (1.040 - 9.250)	0.001
Menopause (reference: no)	1.550 (1.360-1.840)	0.005	1.910 (1.090 - 10.930)	0.001
Fœtal macrosomia (reference: no)	0.020 (0.011-0.041)	<0.001	4.290 (3.320 - 5.550)	<0.001
Vaginal delivery (reference: no)	0.017 (0.002-0.12)	<0.001	2.070 (1.110 - 5.210)	0.006
Vaginal delivery number ≥4 (reference: <4)	6.130 (3.810-9.880)	<0.001	0.170 (0.005 - 5.597)	0.320
Pelvic or perineal tears (reference: no)	0.003 (0.000-0.010)	<0.001	1.510 (1.250 - 1.910)	<0.001

This table presents univariable and multivariable analyses which allowed us to identify factors independently associated with genital prolapse.

## Discussion

The frequency for genital prolapse was of 1.2% at SJH. Our frequency is lower than those found by Hamri´s in Morocco [30], Seven´s in Turkey [31] and Rodrigues in Brazil [32] which were of 2.4%, 5.6% and 7.5% respectively. It is almost identical to those found by Kishawas at Bangladesh (1.1%) [33], Alherrech´s in Morocco (1.1%) [34] and Zhu´s in China (1.2%) [35]. It is higher than 0.5% of Fanny in Ivory Coast [36]. This frequency difference can be explained by the single institution character of our study and the more or less free treatment of genital prolapse in the account of fistula care.

Our study showed that the multiparity (parity≥4) constituted a factor associated with genital prolapse and it multiply significantly the risk of genital prolapse´s appearance by 6. Our observation is in accordance not only with that of Erata who showed that the multiparity is a risk factor for genital prolapse [37], but also with those of many others authors [25-26,38,39]. The occurrence of genital prolapse among multipara is due to the augmentation of pudendal nervous attacks risk (compression and stretching) and muscles direct trauma of pelvic floor (anal levator, anal sphincter, pubo-coccygeal muscles) [27]. These attacks lead to the defect of pelvic floor, root for genital prolapse.

The obesity with body weigth ≥90Kg and BMI ≥30Kg/m^2^was the factor associated with genital prolapse in our study. It augments the risk of genital prolapse by 4 for the body weigth ≥90Kg and by 2 for BMI significantly. Our observation corroborates not only those of Thubert´s [28] and of Mendel´s [40] who noted that the obesity is the factor associated with genital prolapse, but also those of many other authors in the literature [27,41-43]. The role of obesity in the genital prolapse´s occurrence rely on three following arguments: the augmentation of the intra-abdominal pressure which reaches 10.0 more or less 0.6 mmHg at obese people for a normal pressure from 0 to 6 mmHg at non-obese people [27,28,44,45], the augmentation of diabetes mellitus rate complicated of neuropathy at obese person´s, which is at the root of pudendal neuropathy and innervation anomaly of anal levator muscles (Hence, there is absence of their contraction at the rest) [28,46], and the augmentation of vaginal delivery risk for macrosomic newborn at obese person´s [28,47].

The menopause was a factor associated with genital prolapse in our study. It multiply significantly the risk of genital prolapse´s occurrence by 2. Our results are similar to those of many authors in the literature [27,32,38,39]. The role of the menopause in the genital prolapse´s appearance resides in the post-menopausal insufficiency of oestrogen, which provokes modifications of vaginal trophism, of tissue cellularity and of collagen´s metabolism [27,48,49]. Oestrogen´s receptors have been identified to vesical triangle, to urethra, to vaginal mucous membrane, to tendinous arc of perineum, to utero-sacral ligament and to anal levator [27,47-49]. The reduction of oestrogen content provokes the atrophy of these whole tissue which is responsible for the pelvic floor´s weakness. Hence, there is genital prolapse appearance [27,47-49].

Following the example of other studies [26,27,32,37-39], our study showed that the vaginal delivery number≥4, the vaginal delivery and perineal tears constituted factors associated with genital prolapse too. The risk of genital prolapse´s appearance multiplied significantly by 6 in cases of vaginal delivery number≥4. Our results are in the risk interval of genital prolapse´s occurrence according to the vaginal delivery number which is from 3 to 11.5 and described by many other authors in the literature [25,27,37]. Despite their respective OR were of 0.017 and 0.003, the vaginal delivery and perineal tears were significantly associated with genital prolapse´s appearance in our series of cases. The mechanism of genital prolapse´s occurrence in cases of vaginal delivery and perineal tears resides in the risk´s augmentation of pudendal nervous attacks and the degradation of posterior perineal stage thanks to attacks of central fibrous perinal nucleus and anal sphincters. This weakens the pelvic floor, root of genital prolapse [27].

The fœtal macrosomia was the factor associated with genital prolapse. Even if its OR was of 0.021, the fœtal macrosomia was significantly associated with genital prolapse in our series of cases. Our results are in line with those of many other authors in the literature where the fœtal macrosomia was significantly associated with genital prolapse too [25-27,28,37-39,47]. The relation between fœtal macrosomia and genital prolapse is based on the gravity of pelvic floor´s alteration which is secondary to the fœtal macrosomia delivery [27,28].

Factors independently associated with genital prolapse, in our study, were obesity with BMI≥30Kg/m^2^, menopause, fœtal macrosomia, vaginal delivery and perineal tears. Our observation corroborates thoses of many authors in the literature [25,27,28,37-39,46-49]. Weakness of our study is the no evaluation of genetic factors involved in the occurrence of genital prolapse, the no search of immunological tracers for genital prolapse and the non-inference of causality from the associations obtained. Its Strength is the fact that this is the first study on the frequency and risk factors for genital prolapse in hospital milieus of Kinshasa. These data can be used for screening of high-risk women in gynecological and obstetrical consultations in order to improve the treatment of genital prolapse in our milieu.

## Conclusion

Five factors associated with genital prolapse are identifying: obesity with BMI≥30Kg/m^2^, menopause, fœtal macrosomia, vaginal delivery and perineal tears. Our results warrant deepened studies upon genital prolapses in order to allow scientists to raise awareness upon genital prolapse’s studies and to improve its treatment in our milieu. These data could be used for screening of high-risk women in gynecological and obstetrical consultations in the GOS of SJH.

### What is known about this topic

- *The genital prolapse is the dynamic disease which can worsen or recede above all in the pregnant woman's in the postpartum period;*
- *It comprises a great recurrence's risk after surgical treatment and It caused several troubles (urinary, digestive and genital) which hamper the quality of life of patients;*
- *The lack of data on the frequency and risk factors for genital prolapse in hospitals of Kinshasa, in the DR Congo*.

### What this study adds

- *The frequency of genital prolapse is of 1.2%;*
- *Factors associated with genital prolapse include: obesity with BMI≥30Kg/m^2^, menopause, fœtal macrosomia, vaginal delivery and perineal tears;*
- *These results could be used for screening of high-risk women in gynecological and obstetrical consultations*.
